# Endogenous SO_2_ Controls Cell Apoptosis: The State-of-the-Art

**DOI:** 10.3389/fcell.2021.729728

**Published:** 2021-10-07

**Authors:** Yingying Li, Yingjun Feng, Xiaoyun Ye, Hanlin Peng, Jiantong Du, Xiaoli Yao, Yaqian Huang, Hongfang Jin, Junbao Du

**Affiliations:** ^1^Department of Pediatrics, Peking University First Hospital, Beijing, China; ^2^Department of Cardiovascular Medicine, Children’s Hospital Affiliated to Zhengzhou University/Children’s Hospital of Henan Province, Zhengzhou, China; ^3^Department of Ophthalmology, Peking University First Hospital, Beijing, China; ^4^Key Lab of Molecular Cardiology, Ministry of Education, Beijing, China

**Keywords:** sulfur dioxide, metabolism, apoptosis, mechanism, pathophysiology

## Abstract

SO_2_, previously known as the product of industrial waste, has recently been proven to be a novel gasotransmitter in the cardiovascular system. It is endogenously produced from the metabolism pathway of sulfur-containing amino acids in mammalians. Endogenous SO_2_ acts as an important controller in the regulation of many biological processes including cardiovascular physiological and pathophysiological events. Recently, the studies on the regulatory effect of endogenous SO_2_ on cell apoptosis and its pathophysiological significance have attracted great attention. Endogenous SO_2_ can regulate the apoptosis of vascular smooth muscle cells, endothelial cells, cardiomyocytes, neuron, alveolar macrophages, polymorphonuclear neutrophils and retinal photoreceptor cells, which might be involved in the pathogenesis of hypertension, pulmonary hypertension, myocardial injury, brain injury, acute lung injury, and retinal disease. Therefore, in the present study, we described the current findings on how endogenous SO_2_ is generated and metabolized, and we summarized its regulatory effects on cell apoptosis, underlying mechanisms, and pathophysiological relevance.

## Introduction

Previously, SO_2_ was recognized as a water-soluble, colorless, transparent exhaust gas and air pollutant with a sharp odor. High concentrations of SO_2_ in the environment could cause various degrees of damage to humans, animals, plants, and even microorganisms. However, it was discovered that SO_2_ can be endogenously produced in the mammalian metabolism of sulfur-containing amino acids (SAAs) (Stipanuk, 2004; Kimura, 2011). Many studies have shown that endogenous SO_2_ has the unique characteristics of continuous production, rapid diffusion, low molecular weight, and extensive action (Du et al., 2008a; Huang et al., 2016). More interestingly, it was found to play an important role in regulating cardiovascular function and structure under physiologic and pathophysiologic conditions (Du et al., 2008a; Huang et al., 2016). Therefore, we proposed that SO_2_ is a new cardiovascular gaseous signaling molecule. However, the molecular mechanisms underlying its biological action require further studies. It has been found recently that endogenous SO_2_ regulates cell apoptosis, and therefore contributes to the pathogenesis of hypertension, pulmonary hypertension, myocardial injury, brain injury, retinal disease, and acute lung injury (Zhao et al., 2008, 2013, 2019; Wang et al., 2011; Ma et al., 2012; Jin et al., 2013; Han et al., 2014; Du et al., 2018; Liu et al., 2018; Niu et al., 2018; Yang et al., 2018; Zhou et al., 2020). In this article, we described the generation and metabolism of endogenous SO_2_, and summarized the latest findings of the regulation of endogenous SO_2_ on cell apoptosis, its mechanisms, and pathophysiological significance.

## Generation and Metabolism of Endogenous SO_2_

**FIGURE 1:**
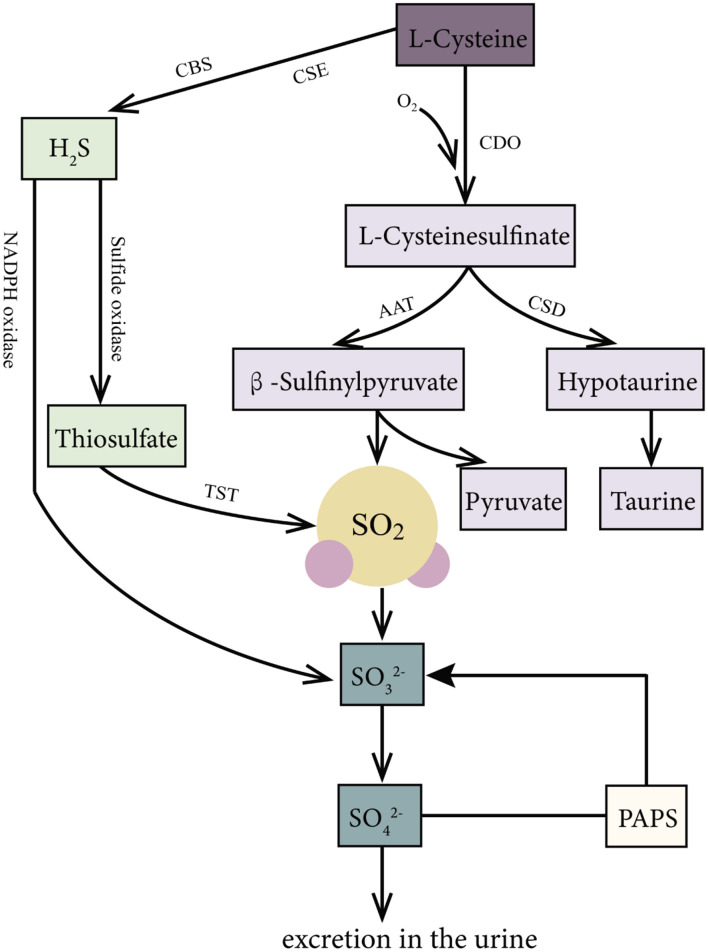
Production and metabolism of endogenous SO_2_ in mammals. L-Cys is metabolized through important enzymes including CDO, AAT, and CSD to generate endogenous SO_2,_ pyruvic acid, and hypotaurine. Moreover, H_2_S, a product of the reaction catalyzed by CSE and CBS with L-Cys as substrate, can be metabolized to sulfite or SO_2_ via the catalyzation of sulfide oxidase, TST, and NADPH oxidase. Thirdly, PAPS in activated neutrophils is also an origin of sulfite. In mammals, SO_2_ can be hydrated to sulfite, then converted into sulfate by sulfite oxidase, and finally excreted in the urine in the kidney. AAT: aspartate aminotransferase; CBS, cystathionine β-synthase; CDO, cysteine dioxygenase; CSE, cystathionine γ-lyase; L-Cys, L-cysteine; NADPH, nicotinamide dinucleotide phosphate; PAPS, 3′-phosphoadenosine-5′-phosphosulfate; TST, thiosulfate sulfurtransferase.

SO_2_ can be produced endogenously through the biotransformation of SAAs in various mammal systems (Figure 1), such as the cardiovascular, respiratory, nervous, digestive, urinary, and immune systems. In the body, SAAs, such as methionine and cystine, are primarily metabolized to L-cysteine (L-Cys). L-Cys is a crucial precursor of endogenous SO_2_ (Singer and Kearney, 1956; Stipanuk, 2004; Du et al., 2008a; Kimura, 2011; Huang et al., 2016). It can be oxidized to produce L-cysteinesulfinate, and this process involves a key enzyme cysteine dioxygenase. Subsequently, the metabolism of L-cysteinesulfinate involves two pathways. In one pathway, L-cysteinesulfinate is converted into β-sulfinylpyruvate via the catalysis of aspartate aminotransferase (AAT), which is followed by spontaneous decomposition of β-sulfinylpyruvate into SO_2_ and pyruvic acid. In the other pathway, L-cysteinesulfinate is decarboxylated into hypotaurine and CO_2_ via cysteinesulfinate decarboxylase. Hypotaurine is subsequently oxidized to taurine. There is a competition between these two pathways for the reaction tendency of L*-*cysteinesulfinate, which is ultimately determined by the activities of AAT and cysteinesulfinate decarboxylase (Singer and Kearney, 1956; Griffith, 1983). Studies have indicated that 3′-phosphoadenosine-5′-phosphosulfate in activated neutrophils functions as an endogenous sulfate donor (Stipanuk, 2004).

Besides, hydrogen sulfide (H_2_S), another gaseous signaling molecule, metabolized from SAAs catalyzed by cystathionine γ*-*lyase or cystathionine β-synthase (Stipanuk, 2004; Kimura, 2011), can be transformed into SO_2_ or sulfite through the following two pathways: (1) H_2_S is oxidized directly to sulfite or SO_2_ through reduced nicotinamide adenine dinucleotide phosphate (NADPH) oxidase (Mitsuhashi et al., 2005). (2) H_2_S is firstly oxidized by sulfide oxidase into thiosulfate, and then the enzymes such as thiosulfate sulfurtransferase or glutathione-dependent thiosulfate reductase catalyze the transformation of thiosulfate to SO_2_ (Shapiro, 1977).

In mammals, SO_2_ can be hydrated to sulfite, and then converted into sulfate by sulfite oxidase, finally excreted in the urine in the kidney (Stipanuk, 2004; Huang et al., 2016). The half-life of SO_2_ was supposed to be about 5–10 min, represented by the fact that serum SO_2_ level decreased by 50% about 5–10 min after a bolus intravenous injection of SO_2_ donor (Du et al., 2008b). It is known that AAT has two isozymes: cytosol AAT1 and mitochondrial AAT2. Huang et al. found that the overexpression of AAT1 or AAT2 increased but the knockdown of AAT1 or AAT2 inhibited the endogenous SO_2_ production, suggesting that AAT1/2 is the main SO_2_-generating enzyme (Liu et al., 2014).

SO_2_ was detected in the headspace gas collected from a closed vial culturing porcine coronary arteries in 2003 by Balazy et al. (2003). Du et al. firstly found the SO_2_/AAT pathway in the cardiovascular tissues of rats in 2008 (Du et al., 2008a). The SO_2_ level in rat plasma was 15.54 ± 1.68 μmol/L while the SO_2_ concentration in the aorta, pulmonary artery, mesenteric artery, tail artery, and renal artery were 3.27 ± 0.21, 2.67 ± 0.17, 2.50 ± 0.20, and 2.23 ± 0.19 μmol/g protein, respectively. To date, SO_2_/AAT pathway has been found in almost all organs in mammals including vessels, heart, stomach, lung, liver, kidney, brain, retina, pancreas, and spleen, etc. (Luo et al., 2011; Du et al., 2018). It was found that the SO_2_ level was the highest in the stomach, followed by the ventricle, and the activity of AAT and the expression of AAT1 mRNA are the highest in the left ventricle, whereas the expression of the AAT1 protein is the highest in the right ventricle. Furthermore, the expression of AAT2 mRNA is the highest in the liver, whereas the expression of the AAT2 protein is the highest in the renal medullary membrane (Luo et al., 2011).

## Mechanisms by Which SO_2_ Regulates Cell Apoptosis and the Significance

Apoptosis is the programmed cell death that occurs in multicellular organisms. Increasing evidence implies that endogenous SO_2_ participates in regulating cell apoptosis (Zhao et al., 2008, 2013, 2019; Wang et al., 2011; Ma et al., 2012; Jin et al., 2013; Han et al., 2014; Du et al., 2018; Liu et al., 2018; Niu et al., 2018; Yang et al., 2018; Zhou et al., 2020, Figure 2). Three main pathways associated with the regulation of SO_2_ on cell apoptosis have been revealed to date: (1) Extrinsic pathway (Wajant, 2002; Zhao et al., 2008): The combination of Fas ligand (FasL)/Fas causes the binding of the Fas-associated death domain proteins which activate caspase-8 precursor (caspase-10 in humans), initiate the cascade activation of caspase-3, caspase-6, and caspase-7 and then induce cell apoptosis. (2) Intrinsic pathway (Jin et al., 2013): The leakage of cytochrome c (Cytc) from impaired mitochondria to cytosol is the central event of the mitochondria-related intrinsic apoptosis pathway. Its preceding events include the deformation and swelling of mitochondria, the imbalance of apoptotic regulator B-cell lymphoma-2 (bcl-2)/bcl-2-associated X protein (bax), the destroy of mitochondrial membrane potential (MMP), and the opening of mitochondrial permeability transition pore (MPTP). The cytosol Cytc activates caspase-9 and caspase-3, finally induces cell apoptosis. (3) Endoplasmic reticulum stress (ERS)-related apoptosis (Wang et al., 2011): The overactivated ERS induces cell apoptosis via overexpressing C/EBP homologous protein (CHOP) and activating caspase-12.

**FIGURE 2:**
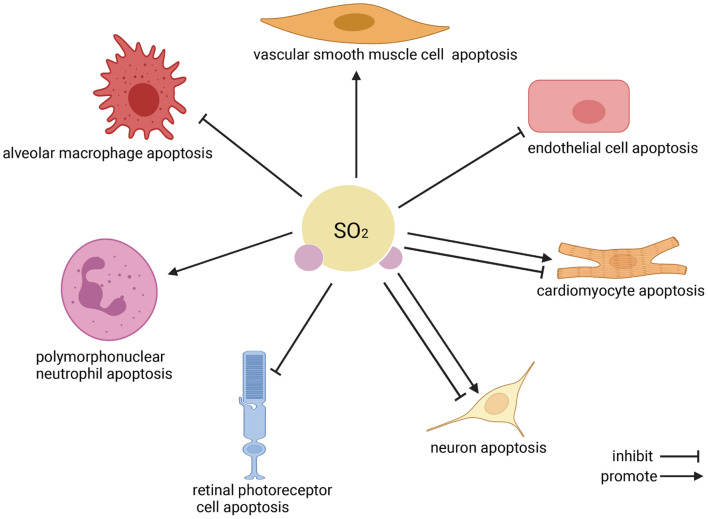
The effect of endogenous SO_2_ on cell apoptosis. According to the clockwise direction, the SO_2_ target cells are shown beginning from vascular smooth muscle cells. In the cardiovascular system, endogenous SO_2_ promotes vascular smooth muscle cell apoptosis, inhibits vascular endothelial cell apoptosis, and inhibits/promotes cardiomyocyte apoptosis. In the nervous system, endogenous SO_2_ inhibits/promotes neuron apoptosis, and reduces the apoptosis of retinal photoreceptor cells. Regarding immune cells, SO_2_ promotes polymorphonuclear neutrophil apoptosis but inhibits macrophage apoptosis.

### Endogenous SO_2_ Promotes Vascular Smooth Muscle Cell Apoptosis

As the main component cell of the vessel, the vascular smooth muscle cell (VSMC) apoptosis markedly affects vascular remodeling. Zhao et al. (2008) found that compared with the WKY rat, the plasma SO_2_ level and the proportion of apoptotic VSMC in media were downregulated in the spontaneously hypertensive rat (SHR). Correspondingly, the blood pressure and the ratio of aorta media to lumen radius were increased by 53.6 and 28.1% in the SHR rat, respectively. Furthermore, SO_2_ donor treatment recovered the decreased plasma SO_2_ content, promoted the VSMC apoptosis, decreased blood pressure, and alleviated the thickening aorta media in the SHR rat. Mechanistically, the expression of antiapoptotic factor bcl-2 was increased but the expression of proapoptotic factor Fas and caspase-3 was decreased in the aorta media in the rats of the SHR + SO_2_ group compared with those in the rats of the SHR group. These data suggested that SO_2_ might alleviate the vascular remodeling by promoting VSMC apoptosis, which involved the regulation of bcl-2, Fas, and caspase-3.

However, SO_2_ did not affect the apoptosis of VSMC with or without fetal bovine serum stimulation *in vitro* (Liu et al., 2014). The discrepant effects of SO_2_ on VMSC apoptosis might be associated with the different experimental conditions such as *in vivo* and *in vitro*. For example, VSMCs are directly challenged by cyclic mechanical stretch and hydrostatic pressure and indirectly affected by endothelial cell (EC)-transduced shear stress under *in vivo* circumstances (Qiu et al., 2013; Chen et al., 2021). The abovementioned biomechanical stresses are reported to control the VSMC apoptosis, proliferation, and other behaviors and only are partly mimicked *in vivo*.

### Endogenous SO_2_ Inhibits Vascular Endothelial Cell Apoptosis

Vascular ECs constitute a huge divider separating the lumen and wall of the vessel, and therefore play a crucial role in maintaining the vascular function and microenvironment homeostasis. Especially, vascular EC apoptosis is a key process involved in the vascular physiological and pathophysiological regulation such as organ development, angiogenesis, vascular injury, and remodeling (Duan et al., 2021; Tisch and Ruiz de Al modóvar, 2021). During the development of hypoxic pulmonary artery structural remodeling, hypoxia-induced vascular EC apoptosis is a critical pathological change. Liu et al. explored the effect of SO_2_ on the human pulmonary artery endothelial cell (HPAEC) apoptosis based on cobalt chloride (CoCl_2_)-stimulated cell model (Liu et al., 2018). The endogenous SO_2_ production and AAT1 expression were down-regulated but the percentage of terminal deoxynucleotidyl transferase-mediated nick end labeling (TUNEL)-positive nuclei was increased in HPAECs of the vehicle + CoCl_2_ group as compared with those of the vehicle group. However, there was no difference in the endogenous SO_2_/AAT1 pathway and apoptosis of HPAECs between the AAT1 overexpression and AAT1 overexpression + CoCl_2_ groups, suggesting that the sufficient endogenous SO_2_ was a powerful antagonist to chemical hypoxia-induced HPAEC apoptosis. Furthermore, AAT1 overexpression prevented the downregulation of antiapoptotic factor bcl-2, and the activation of caspase-3 in the CoCl_2_-treated HPAECs. These data suggested that the endogenous SO_2_ might inhibit the hypoxia-induced HPAEC apoptosis by increasing bcl-2 expression, and inactivating caspase-3.

### Endogenous SO_2_ Regulates Cardiomyocyte Apoptosis

Cardiomyocyte apoptosis is a keystone of cardiac pathological changes and is closely correlated with the development of heart failure, myocardial ischemia-reperfusion (I/R) injury and cardiomyopathy, etc. Endogenous SO_2_ was found to protect against the myocardial I/R, isopropylarterenol (ISO)-induced cardiac injury, sepsis-induced cardiac dysfunction, myocardial infarction, and diabetic cardiomyopathy, suggesting that cardiomyocyte might be an important target cell of endogenous SO_2_ (Liang et al., 2011; Wang et al., 2011, 2018; Chen et al., 2012; Jin et al., 2013; Zhao et al., 2013; Liu et al., 2017; Yang et al., 2018; Zhou et al., 2020).

SO_2_ preconditioning significantly reduced the size of the myocardial infarction and decreased the percentage of TUNEL-positive cells in the rats of myocardial I/R (Wang et al., 2011). The mechanisms by which SO_2_ preconditioning inhibited the cardiomyocyte apoptosis in the myocardial I/R might involve the following (Wang et al., 2011; Zhao et al., 2013): (1) SO_2_ preconditioning induced a moderate ERS, and then inhibited vigorous ERS-initiated cardiomyocyte apoptosis in the development of myocardial I/R injury. The above process was supported by the fact that SO_2_ induced early ERS markers glucose-regulated protein 78 (Grp78) expression and eukaryotic initiation factor 2α (eIF2α) phosphorylation, while myocardial I/R induced early and late ERS markers including Grp78 expression, eIF2α phosphorylation, CHOP expression and caspase-12 activation, which could be alleviated by SO_2_ preconditioning. (2) SO_2_ preconditioning inhibited the activation of caspase-9 and caspase-3 in the rats of the myocardial I/R group. Considering that caspase-9 activation subsequently follows the leakage of cytosol Cytc from injury mitochondria, the antiapoptotic effect of SO_2_ preconditioning might involve mitochondrial protection. (3) The inhibitor of PI3K/Akt the pathway blocked the SO_2_ preconditioning-inhibited caspase-3 activation, suggesting that the activation of the PI3K/Akt pathway might mediate the protective role of SO_2_ preconditioning.

The effect of endogenous SO_2_ on the ISO-induced cardiomyocyte apoptosis was examined targeting the mitochondrion (Liang et al., 2011; Jin et al., 2013). The SO_2_ level in the myocardial tissue was decreased but the proportion of apoptotic cells was increased in the rats of the ISO group compared with those of the control group. SO_2_ treatment restored the myocardial SO_2_ level and inhibited cardiomyocyte apoptosis. Then, the study further focused on the mitochondria. Firstly, the stereological analysis of the mitochondria showed that SO_2_ alleviated the cardiomyocytic mitochondria swelling and deformation, represented by the fact that SO_2_ treatment decreased the mitochondrial mean surface area, mean volume and volume density, and increased the mitochondrial numerical density and surface-to-volume ratio in the myocardial tissue of ISO-treated rats. Secondly, SO_2_ upregulated bcl-2 expression and downregulated bax expression, improved the impaired MMP, and reclosed the MPTP in rat myocardial tissues. Subsequently, SO_2_ blocked the leakage of Cytc from the mitochondria and inhibited the activation of cytosol caspase-9 and caspase-3. These data suggested that endogenous SO_2_ prevented ISO-induced cardiomyocytic apoptosis via maintaining the mitochondrial function and structure. Moreover, SO_2_ decreased the protein and mRNA levels of ERS markers Grp78, CHOP, and caspase-12 in the myocardial tissues of ISO-treated rats, suggesting that the over-activated ERS might also be involved in the inhibitory effect of SO_2_ on the ISO-induced cardiomyocytic apoptosis (Chen et al., 2012).

Similar to the ISO-induced myocardial injury, SO_2_ treatment inhibited cardiomyocytic apoptosis in the myocardial tissue of rats with cecal ligation and puncture (CLP), accompanied by an increase in the bcl-2 expression, and a decrease in the bax/bcl-2 ratio and caspase-3 activation (Yang et al., 2018). Moreover, SO_2_ decreased the level of inflammatory cytokines, inhibited TLR4 and NLRP3 expression, and reduced the inflammatory caspase-1 expression in the myocardial tissue of CLP rats and the LPS-treated primary neonatal rat cardiac ventricular myocytes (Yang et al., 2018), suggesting that NLRP3/caspase-1-mediated cell pyroptosis might also participate in the protective role of SO_2_ on the sepsis-induced cardiac dysfunction.

In some studies, SO_2_ promoted cardiomyocytic apoptosis *in vitro* and *in vivo* (Li et al., 2019). The data showed that the SO_2_ increased the proportion of apoptotic cells, activated caspase-9 and caspase-3, destroyed the MMP, reduced bcl-2 expression and the ratio of bcl-2/bax, and upregulated the expression of bax and p53, which could be blocked by antioxidant N-acetylcysteine. We speculated the following explanations for the different effects of SO_2_ on cardiomyocyte apoptosis. (1) The experimental reagents were different. Li et al. used sodium bisulfite solution and SO_2_ gas inhalation *in vitro* and *in vivo*, respectively, while a mixed solution of sodium sulfite and sodium bisulfite with a molar ratio of 3:1 was used in other studies. (2) The experimental conditions were different. Li et al. used untreated cardiomyocyte H9c2 cells and rats, while other studies were conducted on the different pathological cell and rat models.

### Endogenous SO_2_ Regulates Neuronal Apoptosis

The relationship between endogenous SO_2_ and neuronal apoptosis in the neurological disease was investigated on the kainic acid (KA)-induced epilepsy, febrile seizure (FS), and transient global ischemia/reperfusion models (Han et al., 2014; Niu et al., 2018; Zare Mehrjerdi et al., 2018). The different effects of SO_2_ on neuronal apoptosis depended on the neurological disorders and SO_2_ donor concentrations.

In the KA-induced epileptic rats, plasma SO_2_ level and AAT activity were upregulated compared with those in control rats (Niu et al., 2018). However, an AAT inhibitor HDX treatment retarded the occurrence of hippocampal neuronal apoptosis in the KA-treated rats, represented by the fact that a significant hippocampal neuronal apoptosis was detected in the rats 72 h after treatment with KA, while it was not observed in the rats treated with KA + HDX until 7 days after the intervention. The results suggested that the upregulated endogenous SO_2_ might promote hippocampal neuronal apoptosis in the development of epilepsy-related brain injury. Furthermore, the KA-upregulated ERS markers Grp78 expression and phosphorylated PERK in the hippocampus could be inhibited by HDX, indicating that ERS might participate in the promotive effect of SO_2_ on hippocampal neuronal apoptosis.

The endogenous SO_2_/AAT pathway including SO_2_ content and AAT1/2 expressions was upregulated in the hippocampus in rats of recurrent FS, accompanied by an increase in the neuronal apoptosis and mossy fiber sprouting (MFS) in the hippocampus (Han et al., 2014). Furthermore, HDX treatment aggravated the increased neuronal apoptosis and MFS in recurrent FS rats, while the supplement of a low concentration of SO_2_ (1–10 μmol/kg) alleviated the above pathological change, suggesting that the upregulated endogenous SO_2_ might be a defensive mechanism for antagonizing the neuronal apoptosis in the development of recurrent FS. However, the supplement of a high concentration of SO_2_ donor (100 μmol/kg) worsened the neuronal apoptosis and MFS in recurrent FS rats, which reminded us to pay attention to the protective dose range of SO_2_.

A global brain I/R injury increased CA1 neuronal apoptosis in the rats undergoing the occlusion of both common carotid arteries, accompanied by an increase in malondialdehyde level, and a decrease in glutathione level and activity of superoxide dismutase in the CA1 region (Zare Mehrjerdi et al., 2018). Furthermore, SO_2_ donor treatment improved the learning and memory deficits in the rats with cerebral I/R injury, reduced CA1 neuronal apoptosis, decreased malondialdehyde level, and elevated GSH level and activity of superoxide dismutase in the CA1 region. These findings indicated that SO_2_ donor prevented brain ischemic injury by decreasing neuronal apoptosis and the underlying mechanisms might involve the anti-oxidative effect of SO_2_. However, the change of endogenous SO_2_/AAT pathway in the brain ischemic rats was not detected, which merits further studies for exploring the role of endogenous SO_2_ in the development of brain ischemic injury.

### Endogenous SO_2_ Inhibits Retinal Photoreceptor Cell Apoptosis

Oxidative stress can induce apoptosis of retinal photoreceptor cells, which is the main pathological change of blinding retinal diseases (Wenzel et al., 2005). Du et al. found that apoptosis was significantly induced in the 661w mouse photoreceptor cells exposed to H_2_O_2_ compared with those without H_2_O_2_ exposure, demonstrated by an increase in the percentage of TUNEL positive cells and the ratio of cleaved-caspase3/caspase3 (Du et al., 2018). Simultaneously, endogenous SO_2_ production and AAT1 expression were decreased in the H_2_O_2_-treated 661w cells compared with control cells. HDX treatment mimicked the effects of H_2_O_2_ on 661w cells including an increase in the cell apoptosis and the downregulation of endogenous SO_2_/AAT pathway, indicating that the apoptosis of 661W cells caused by oxidative stress might be mediated by the downregulation of the endogenous SO_2_ pathway. However, the role of endogenous SO_2_ in the development of retinal photoreceptor cell apoptosis and its mechanisms require further studies.

### Endogenous SO_2_ Promotes Polymorphonuclear Neutrophil Apoptosis

Lipopolysaccharide (LPS) was intratracheally instilled to induce acute lung injury (ALI) in adult male rats (Huang et al., 2009). The decrease in the apoptosis of polymorphonuclear neutrophils (PMNs) contribute to an increment of the number of PMN and a prolonged inflammatory reaction in the injured lung (Lee and Downey, 2001). LPS treatment reduced the SO_2_ level in the lung tissue and peripheral blood, while the treatment of SO_2_ donor alleviated the lung histopathological changes in the rats accompanied by a reduction of PMN number in the bronchoalveolar lavage fluid (Huang et al., 2009; Ma et al., 2012). Furthermore, SO_2_ promoted the apoptosis of PMN in the bronchoalveolar lavage fluid and peripheral blood in the ALI rats. Mechanistically, SO_2_ treatment upregulated the expression of bax, downregulated the expression of bcl-2, and elevated the level of activated caspase-3 in the peripheral PMN in the ALI rats. Therefore, it is believed that SO_2_ may antagonize ALI by promoting PMN apoptosis, in which the bax/bcl-2/caspase-3 pathway is involved.

### Endogenous SO_2_ Inhibits Alveolar Macrophage Apoptosis

Many studies focused on the role of alveolar macrophage (AM) in the pathogenesis of ALI (Cheng et al., 2021). It was found that AM apoptosis was significantly increased in the ALI rat model induced by limb I/R injury, resulting in the delayed clearance of inflammatory PMN and the prolongation of ALI (Zhao et al., 2016). Zhao et al. found that SO_2_ donor prevented limb I/R-induced ALI in rats (Zhao et al., 2016). Furthermore, the primary AM was cultured with the plasma extracted from limb I/R to make the limb I/R-induced AM injury model (Zhao et al., 2019). Compared with the ALI group, the AM apoptosis was reduced in the AMs of the ALI + SO_2_ group, with an increase in bcl-2 expression, an improvement of the impaired MMP, the reclosing of MPTP and the decrease in caspase-3 expression. These data suggested that the SO_2_-inhibited AM apoptosis might be involved in the protective effect of SO_2_ on ALI and mitochondrion was the possible target of SO_2_.

## Conclusion and Perspective

As a novel gaseous signaling molecule, endogenous SO_2_ was reported to play a variety of biological effects by regulating cell apoptosis. In summary, endogenous SO_2_ promoted VSMC apoptosis and inhibited vascular EC apoptosis to alleviate the vascular remodeling in hypertension and pulmonary hypertension. Endogenous SO_2_ suppressed the apoptosis of cardiomyocytes, contributing to the cardioprotective effect of SO_2_. In the nervous system, SO_2_ increased KA-induced hippocampal neuronal apoptosis to aggravate the epileptic brain damage, while SO_2_ reduced neuronal apoptosis to protect against the neuronal damage in the recurrent FS and global brain I/R injury. Moreover, the downregulation of endogenous SO_2_ was accompanied by the increase in the 661w retinal photoreceptor cell apoptosis, suggesting that endogenous SO_2_ might participate in the pathogenesis of blinding retinal disease. In the respiratory system, SO_2_ facilitated PMN apoptosis and inhibited AM apoptosis to protect against ALI. The abovementioned apoptosis-related studies proved that the abnormal endogenous SO_2_ was involved in the pathogenesis of many diseases such as cardiovascular disease, neurological disease, and respiratory disease, and then keeping endogenous SO_2_ in the normal range might be a new and meaningful therapeutic strategy of related diseases in the future.

However, there remain some issues to be taken into account in future studies: (1) The endogenous SO_2_ generation and its producing enzyme should be firstly detected in the animal and cell models to identify the role of endogenous SO_2_ in the pathogenesis of diseases. (2) The concentration and type of SO_2_ donors might affect the effect of SO_2_, which should be paid attention to *in vitro* and *in vivo*. (3) Since mitochondria and their related apoptosis pathways were reported to be involved in almost all the anti- or pro-apoptotic effects of endogenous SO_2_, the detailed target molecules, and mechanisms by which SO_2_ affects mitochondria merit expectation in the future. (4) A recent study showed useful findings that plasma SO_2_ as a practical biomarker could predict the occurrence of acute kidney injury in the patients staying in the surgical intensive care unit (Jiang et al., 2021). Therefore, as a biomarker, the level of SO_2_ in clinical samples might have translational potential in the diagnosis, treatment decision, and prognosis prediction of the diseases. (5) In the future, the SO_2_-released prodrug and AAT inhibitor in clinical application for apoptosis-related diseases are expected to further studies (Day et al., 2016; Wang and Wang, 2018; Huang et al., 2021).

## Author Contributions

All authors listed have made a substantial, direct and intellectual contribution to the work, and approved it for publication.

## Conflict of Interest

The authors declare that the research was conducted in the absence of any commercial or financial relationships that could be construed as a potential conflict of interest.

## Publisher’s Note

All claims expressed in this article are solely those of the authors and do not necessarily represent those of their affiliated organizations, or those of the publisher, the editors and the reviewers. Any product that may be evaluated in this article, or claim that may be made by its manufacturer, is not guaranteed or endorsed by the publisher.
